# Predictors of Depression in Healthcare Patients at Risk of Self-Neglect

**DOI:** 10.1093/geroni/igab046.1061

**Published:** 2021-12-17

**Authors:** Miriam Rose, Farida Ejaz, Courtney Reynolds

**Affiliations:** 1 Benjamin Rose Institute on Aging, Cleveland, Ohio, United States; 2 Benjamin Rose Institute on Aging, Benjamin Rose Institute on Aging, Ohio, United States

## Abstract

More than half of reports to Adult Protective Services agencies nationwide involve allegations of self-neglect. An intensive case management intervention for preventing self-neglect was evaluated in a longitudinal study conducted collaboratively by a large healthcare system, Adult Protective Services, and a gerontological research institute. Patients (444) who were older (60+ years) and/or disabled (18+ years) were randomly selected for participation from 19 primary-care clinics if they had risk factors for self-neglect, including depression, substance abuse, dementia, and/or impairment in activities of daily living. Average age was 68 years (SD=12.5), 68% were Hispanic, 68% had monthly income of less than $1,361, and 67% were female. Clinics were randomized into intervention and control groups. Intervention clinic patients received intensive case management services; control clinic patients received usual care, including social work services. Subjects were interviewed at baseline and four months later. The Stress Process Model guided a multiple regression analysis. Domains of background characteristics, primary and secondary stressors, and support (patients in intervention or control group) were entered in blocks to predict depression levels at post-test. While no significant differences were found in post-test depression levels between intervention and controls, the final model was statistically significant (adjusted R2=.452). Significant predictors of depression were: younger age (disabled adults), poorer self-rated physical and emotional health, greater loneliness, and less social support. Future analyses will examine effects of moderating variables on post-test depression levels. Practice implications of preliminary analyses include addressing disabled adults’ mental health needs, especially if they are isolated and lack social support.

